# Distribution of *Kazachstania* Yeast in Thai Traditional Fermented Fish (Plaa-Som) in Northeastern Thailand

**DOI:** 10.3390/jof8101029

**Published:** 2022-09-28

**Authors:** Sukrita Punyauppa-path, Pongpat Kiatprasert, Prasongsom Punyauppa-path, Pongsak Rattanachaikunsopon, Pannida Khunnamwong, Savitree Limtong, Nantana Srisuk

**Affiliations:** 1 Department of Mathematics and Science, Faculty of Agriculture and Technology, Rajamangala University of Technology, Isan Surin Campus, Surin 32000, Thailand; 2 Department of Biological Science, Faculty of Science, Ubon Ratchathani University, Warin Chamrap District, Ubon Ratchathani 34190, Thailand; 3 Department of Microbiology, Faculty of Science, Kasetsart University, Bangkok 10900, Thailand; 4 Biodiversity Center Kasetsart University (BDCKU), Bangkok 10900, Thailand; 5 Academy of Science, The Royal Society of Thailand, Bangkok 10300, Thailand

**Keywords:** fermented food, fermented fish, *Kazachstania*, Plaa-som, Thai fermented fish

## Abstract

Thai traditional fermented fish products (Plaa-som) from four provinces (Ubon Ratchathani, Surin, Sisaket, and Khon Kaen) in the northeast part of Thailand were collected and analyzed to determine their salt content, total acidity, and pH. Yeasts in all samples were isolated and identified to the genus and species level based on sequence analysis of the D1/D2 of the large subunit (LSU) rRNA gene and the internal transcribed spacer (ITS) region. The results revealed that the salt content, total acidity, and pH values are in the range of 2.01–6.9%, 0.62–1.9%, and 4.4–6.57%, respectively. Moreover, 35 strains of yeast were isolated and identified as eight genera, namely *Candida*, *Diutina, Filobasidium, Kazachstania, Pichia, Saccharomyces, Torulaspora*, and *Yarrowia* with 17 species. The ascosporogenous yeast, *Kazachstania*, was the most dominant genus found and was widely distributed among the fermented food samples. In addition, a new strain of yeast, *Kazachstania surinensis*, was also discovered in Plaa-som samples. Thus, this study is the first to report the presence and wide distribution of these yeasts in fish fermentation products.

## 1. Introduction

Fermented food is a Thai folk wisdom that has been passed down for hundreds of years. The production of Thai traditional fermented foods has been trial and error without any scientific support. Therefore, most of them, if not all, have depended on natural fermentation or spontaneous fermentation leading to heterogeneous microbiota in the final product [1].

Plaa-som is a Thai traditional fermented fish, obtained from a fermentation of fish with salt, steamed rice, and chopped garlic via a solid-state fermentation for 3 to 7 days [2,3]. Plaa-som can be made by using whole fish (Plaa-som), fish fillet (Plaa-som pieces), fish strips (Plaa-som strip), and fish mince (Plaa-som Fak). The fish commonly used for making Plaa-som in the Northeast of Thailand is freshwater fish such as silver barb (*Barbus gonionotus*), Siamese mud carp (*Henicorhynchus siamensis*), and small scall mud carp (*Cirrhinus microlepis*) [2,3].

Production of Plaa-som is done in a spontaneous fermentation via traditional methods that have been passed down from generation to generation [4,5]. The amount of salt, rice, and garlic in Plaa-som production ranged from 2–11%, 1–5%, and 3–5% (*w*/*w*), respectively, but the ratio used in production varies from area to area [6,7,8]. In addition, Plaa-som also has ingredients (fish, salt, rice) and preparation methods that resemble other traditional fermented fish such as Plaa-paeng-dang (Thailand), Bu-rong isda (Philippine), Mam chua (Vietnam), Bekasem (Indonesia), and Narezushi (Japan) [9,10,11].

The fermentation process in traditional fermented foods can, in fact, basically be performed either by spontaneous fermentation, by back-slopping, and by adding starter cultures. In spontaneous fermentation, indigenous microorganisms come from raw materials, utensils, containers, and environments together with optimal conditions to select and promote the growth of microorganisms that cause fermentation in foods. Back-slopping is a method of using part of the fermented product from the previous batch as the inoculum for the new batch, while the use of pure culture as the inoculum is done in local fermented foods that are produced on an industrial scale [9].

In traditional fermented foods, spontaneous fermentations often involve interactions between microbial groups, such as bacteria-bacteria, yeast-yeast, and yeast-bacteria. These interactions create a group of heterogeneous microorganisms that work synergistically, resulting in a significant impact on taste, texture, and odor [12,13]. The two main groups of microorganisms that play an important role in creating the distinctive characteristics of fermented fish are lactic acid bacteria and yeast [6,14]. Lactic acid bacteria (LAB) and yeasts have been known to play major roles in food fermentation by acting in cooperation to form a complex microbiota. Up until now, lactic acid bacteria have been in the spotlight as the main microorganisms in food fermentation because they produce many substances such as organic acids, aromatic compounds, and peptides not only to drive the fermentation process but also to inhibit growth of undesirable organisms.

Since lactic acid bacteria is considered as the majority of bacteria in fish fermentation, previous research focused on the lactic acid bacteria rather than yeast. Therefore, many genera and species of lactic acid bacteria in various types of fermented foods such as *Pediococcus pentosaceus*, *P. halophilus*, *Lactobacillus alimentarius/farciminis*, *L. plantarum*, *L. pentosus*, *Weisella onfuse*, *Streptococcus bovis*, and *Lactococcus garviae* were screened, isolated, and identified [2,6,15,16,17,18,19,20]. There are only few research reports of the dominant yeast species in these types of fermented foods. Furthermore, the dominant yeast generally found in these fermented foods (fish and meat) were of the genera *Candida*, *Saccharomyces*, and *Pichia* with some *Zygosaccharomyces*, *Debaryomyces*, and *Hanseniaspora* [6,21,22]. We present here the findings of the most dominant and widely distributed genus of yeast (*Kazachstania*) among the 35 samples of Thai fermented fish collected from Northeastern Thailand.

## 2. Materials and Methods

### 2.1. Collection of Plaa-Som Samples

Fermented fish samples (Plaa-som) were collected from selected locations in four different provinces, Ubon Ratchathani, Surin, Sisaket, and Khon Kaen, in the northeastern part of Thailand (Table 1). Samples were purchased, kept in a foam box with ice, and immediately transported to the laboratory and kept refrigerated (4 °C) until use.

### 2.2. Chemical Analyses

#### 2.2.1. Measurement of pH

The pH of the samples was determined using 2 g of homogenized samples mixed with 20 mL of carbon-dioxide-free distilled water. Direct pH measurement was done using a standard pH meter (S20 SevenEasy™, Mettler-Toledo, Inc., Columbus, OH, USA). Triplicate determinations of each treatment were performed.

#### 2.2.2. Determination of Total Acidity

The total acidity of the sample was determined according to AOAC standard methods [23]. A 2 g sample was homogenized in 20 mL of carbon-dioxide-free distilled water using a homogenizer (Ultra-Turrax, IKA Labortechnik, Staufen, and Germany). The homogenate was then centrifuged at 5000× *g* for 15 min. Then, the supernatant was filtered through Whatman No. 4 filter paper (Whatman International Ltd., Maidstone, UK). Three drops of a phenolphthalein solution (1% *w*/*v*) were added to the filtrate and titrated with a standardized 0.1 N NaOH solution until a light pink color was observed. The total acidity was calculated as an equivalent to lactic acid and reported as a percentage (*w*/*w*). Triplicate determinations on each treatment were performed.

#### 2.2.3. Determination of Salt Content

The percentage of salt in the samples was determined according to a procedure described by AOAC International [24] and Kopermsub and Yunchalard [2]. Five grams of a homogenized sample were accurately weighed and mixed with 25 mL of distilled water. The sample was then filtered through Whatman No. 4 filter paper (GE Healthcare Thailand, Bangkok, Thailand). The filtrate was titrated using 0.1 N silver nitrate with an addition of 1 mL of 0.25 M potassium chromate as an indicator. Salt content was calculated from the volume of 0.1 M silver nitrate used to reach the end point of the titration and reported as %sodium chloride (*w*/*w*). Triplicate determinations on each treatment were performed.

#### 2.2.4. Yeast Isolation

Ten grams of each Plaa-som sample were homogenized in 90 mL of 0.85% (*w*/*v*) NaCl and tenfold serial dilutions were prepared until the desired concentrations were obtained. One hundred microliters of each dilution (10^−3^ to 10^−5^ dilutes) was spread onto yeast extract peptone dextrose agar (YPD) supplemented with 0.25% (*w*/*v*) sodium propionate. All agar plates were incubated at 25 °C for 1–2 days. Colonies were randomly isolated from the highest dilution on YPD agar. Representative yeast colonies were picked based on colony characteristics and purified using a cross-streaking method on YPD agar. Purified yeast cultures were preserved in YPD broth supplemented with 20% (*v*/*v*) glycerol at −20 °C [21,25].

#### 2.2.5. Yeast Identification

The methods used for DNA extraction from whole yeast cells and amplification were described by Limtong et al. [26]. Amplification of the D1/D2 region of the LSU rRNA gene was done using PCR with the forward primer NL1-F (5′-GCA TATCAATAAGCGGGGAAAAG-3′) and the reverse primer NL4-R (5′-GGTCCGTGTTTCAAGACGG-3′) [27]. The ITS region was amplified with the forward primer ITS1-F (5′-TCCGTAGGTGAACCTGCGG-3′) and the reverse primer ITS4-R (5′-TCCTCCGCTTATTGATATGC-3′) [28]. The sequences were compared with available sequence data using a BLASTN search in the NCBI GenBank database [29]. They were aligned with sequences of related species retrieved from GenBank using the program, MEGA Version 7 [30]. The phylogenetic distances of the yeast species were calculated based on the maximum-likelihood method, applying the general time-reversible (GTR) model and using the concatenated ITS and D1/D2 sequences. Confidence values of the branch node were evaluated from bootstrap analysis (1000 replicates) [31].

#### 2.2.6. Yeast Community Analysis

The Shannon–Wiener index (*H’*) and radar chart were used to determine the diversity of yeast communities. The following formulas were used:

Shannon–Wiener index

(1) $$H=-\sum_{i=0}^{s}Pi\;(\ln Pi)$$

where *Pi* is the proportion of each species in the sample and S is the total number of species in the total sample [32].

The relative frequency (%) was calculated as the number of yeast strains of an individual species as a proportion of the total number of yeast strains. The frequency of occurrence (%) was calculated as the number of samples, where a particular genus was observed, as a proportion of the total number of samples.

## 3. Results

### 3.1. Collection of Plaa-Som Samples

In this study, 35 Plaa-som samples were collected from five fresh markets in Ubon Ratchathani, Surin, Sisaket, and Khon Kaen provinces. The location of each market is shown in Table 1.

### 3.2. Determination of Salt Content

**Figure 1:**
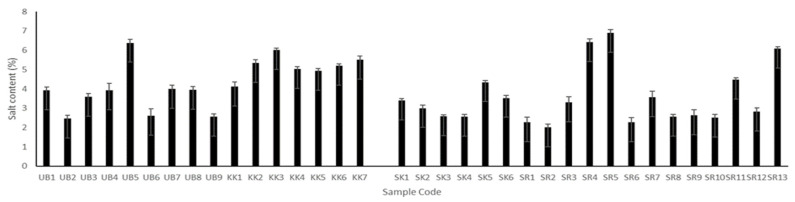
Salt content of Plaa-som samples from four provinces: Ubon Ratchathani (UB), Khon Kaen (KK), Sisaket (SK), and Surin (SR).

The salt contents of all Plaa-som samples were different regardless of their origin (as shown in Figure 1). Fermented samples contained salt in the range of 2.01% to 6.9%. However, samples with the lowest and the highest salt contents came from Surin province (SR2, 2.01% and SR5, 6.9%). It was also found that the salt content of Plaa-som from Sisaket and Khon Kaen varied over narrow ranges of 2–4% and 4–6%, respectively, while those from Ubon Ratchathani and Surin presented higher variations. Plaa-som products are commonly produced according to family tradition and local geographic preferences. The various salt levels of the Plaa-som samples result from the recipes used, which have been passed down from generation to generation. This creates the distinctive taste and texture of fermented fish products from each area.

The salt content of Plaa-som has been studied by several researchers. It was found to vary from 1.4–11% [2,6,15,33]. The salt used in Plaa-som not only serves as a seasoning, but also acts to establish optimal conditions for the growth of microorganisms that play a role in fish fermentation. As a seasoning, it has been found that a high salt content can hide real delicate aromas and flavors of fermented food products. However, with a low salt content, there is less of a microbial barrier against the growth of undesirable micro-organisms in these food products [2,34,35]. In the case of creating optimal conditions for the growth of microorganisms, an inappropriate salt content can affect the type and number of the microorganisms that play important roles in fermented fish processes. Paludan-Müller [36] and Paludan-Müller et al. [6] found that increasing salt concentrations from 6% to 11% delayed LAB growth and thereby the fermentation process of Plaa-som. The optimum salt content is in the range of 6–7% in order to facilitate the growth of lactic acid bacteria, resulting in a lower pH, less than 4.5, that is suitable for the growth of yeast in the Plaa-som fermentation process [17,37].

### 3.3. Determination of pH

**Figure 2:**
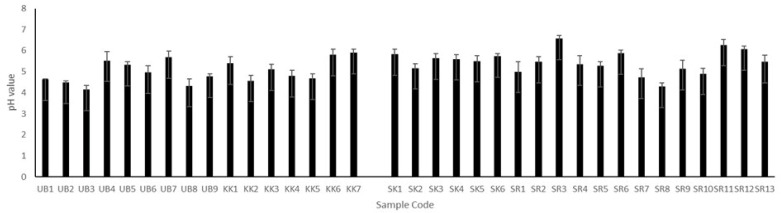
The pH values of Plaa-som samples from 4 provinces: Ubon Ratchathani (UB), Khon Kaen (KK), Sisaket (SK), and Surin (SR).

It was found that the pH of Plaa-som varied over the range of 4.14–6.57 (as shown in Figure 2). These results are consistent with numerous published papers reporting that the pH of Plaa-som ranged from 3.2–6.4 [2,6,15,17,33,36,38].

In general, the pH of Plaa-som depends on three factors: the salt content, amount of carbohydrates utilized in the fermented fish, and the fermentation time. Details of these factors are given below.

The salt concentration can affect the growth of microorganisms involved in the fermentation process, particularly lactic acid bacteria. An appropriate salt content promotes the growth of lactic acid bacteria, resulting in low pH, whereas a high salt content inhibits their growth, resulting in higher pH values [6,34,36,37,39].

The carbohydrate level in Plaa-som is the second factor that affects the pH of Plaa-som. Lactic acid bacteria in Plaa-som use added carbohydrates as a carbon source and produce the major metabolite, lactic acid [14,15,39,40]. Therefore, a high carbohydrate content together with the growth of lactic acid bacteria can cause a high acid content, resulting in low pH values [33,34,37].

The final factor influencing the pH of Plaa-som is the fermentation time. Kopermsub and Yunchalard [2] followed the fermentation of fish for 6 to 144 h and found that the pH of Plaa-som samples decreases with increasing fermentation time. Long fermentation times resulted in high levels of lactic bacteria, leading to the formation and accumulation of acid in the Plaa-som, resulting in lower pH values in Plaa-som samples.

Since the salt contents of our samples were less than 7% (2.01 to 6.9%), all Plaa-som samples are categorized to low-salt fermented fish. These salt contents correspond to the optimal salt content, 6–7%, suitable for the growth of lactic acid bacteria [6,36]. Additionally, our pH measurements of Plaa-som samples ranged from 4.14–6.57, which is similar to values reported in previously published papers [2,6,15,17,36,38]. Unfortunately, we do not have details of the initial carbohydrate content and fermentation time of each Plaa-som sample. The sellers are not Plaa-som manufacturers, so they do not know this information. Therefore, the amount of carbohydrates and fermentation time cannot be discussed concerning the pH of Plaa-som samples in this study.

### 3.4. Determination of Total Acidity

**Figure 3:**
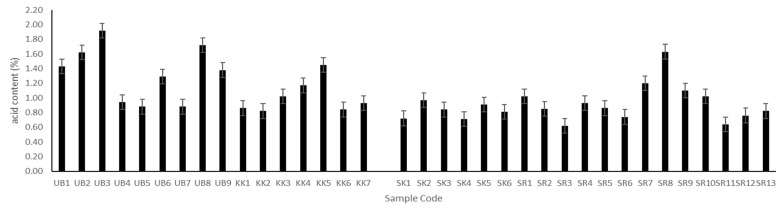
Acid content of Plaa-som samples from 4 provinces: Ubon Ratchathani (UB), Khon Kaen (KK), Sisaket (SK), and Surin (SR).

The main factors contributing to acid production in Plaa-som are lactic bacteria, carbohydrate content, and fermentation period. In the current study, the total acidity of Plaa-som samples ranged from 0.62–1.92% (Figure 3), which is in agreement with previously published papers [2,6,36,38]. The lowest acid content sample, SR2 (pH 6.57), was from Surin province, while the highest acid sample was UB3 (pH 4.14) from Ubon Ratchathani. Although pH values of all Plaa-som samples were highly variable (4.14–6.57), the pH values of Plaa-som did not fluctuate widely due to the high buffering capacity of fish muscle protein. This kept the pH from becoming too low during the late stages of the fermentation [2,36].

### 3.5. Yeast Recovery and Isolation

Thirty-five Plaa-som samples (*n* = 35) were purchased from different food sellers at five fresh markets in Ubon Ratchathani, (*n* = 9), Surin (*n* = 13), Sisaket (*n* = 6), and Khon Kaen (*n* = 7). Yeasts in each sample were isolated using the methodology described above. The recovery rates of yeasts from Plaa-som samples collected from Sisaket, Khon Kaen, Surin, and Ubon Ratchathani were 83.33%, 71.43%, 46.15%, and 44.44%, respectively. The overall percentage of samples in which yeast were detected was 57.14% (Table 2). Yeast colonies with a distinctive appearance were purified using a cross-streak technique on malt yeast extract agar (MYA) and then examined under a microscope before making stock cultures on MYA slants [41,42].

### 3.6. Yeast Identification

**Figure 4:**
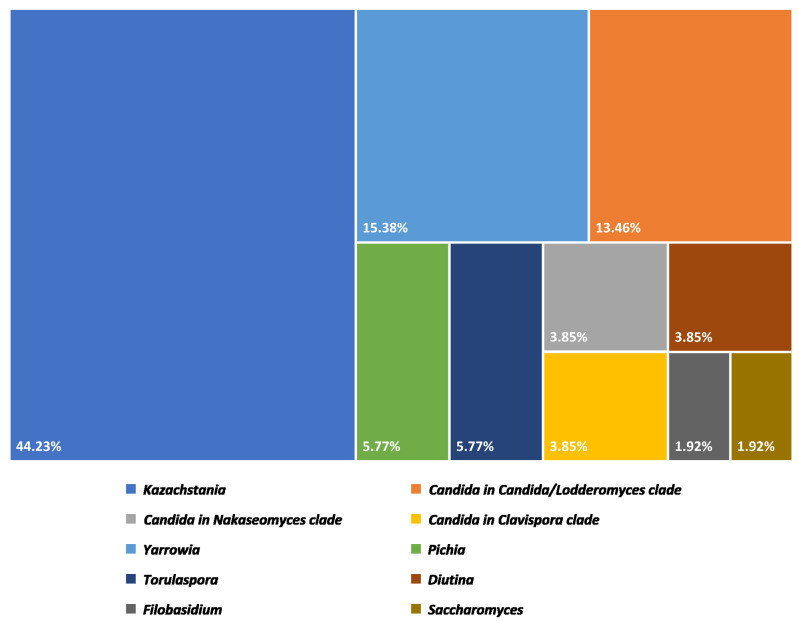
Proportion of yeast genera discovered in Plaa-som samples collected from fresh markets in Thailand.

Fifty-two isolates of yeast from Plaa-som samples were identified into eight genera and seventeen species. The detail and proportion of yeast genera in Plaa-som samples are shown in Figure 4.

**Figure 5:**
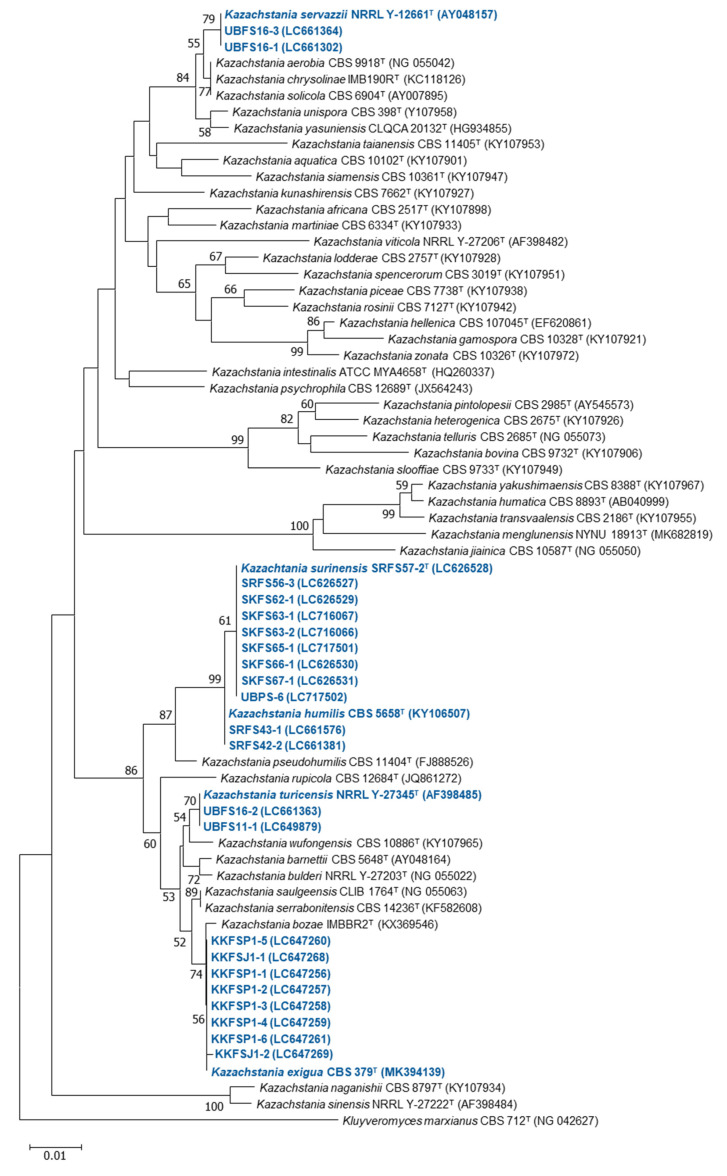
A neighbor-joining phylogenetic tree of *Kazachstania* yeasts isolated from Plaa-som samples in this study (blue) and all other recognized *Kazachstania* species (black) based on the D1/D2 region of the LSU rRNA sequence data set. The bootstep values are calculated using 1000 re-samplings for indicating the nodes. It should be noted that strains SKF62-1, SKF66-1, SKF67-1, SRF56-3, and SRF57-2 were described as *Kazachstania surinensis* [43]. Strains SKF63-1, SKF63-2, SKF65-1, and UBPS-6 were also identified as *Kazachstania surinensis*.

The number of yeast and relative frequency of each species is presented in Table 3. Identification to the species level was done with a molecular method using analysis of the D1/D2 domain (shown in Table 4) and neighbor-joining phylogenetic analysis of *Kazachstania* strains (shown in Figure 5).

A phylogenetic tree constructed using the neighbor-joining method showing the position of strains and related yeast species based on 28S rDNA sequences and the bootstrap consensus tree inferred from 1000 replicates.

**Figure 6:**
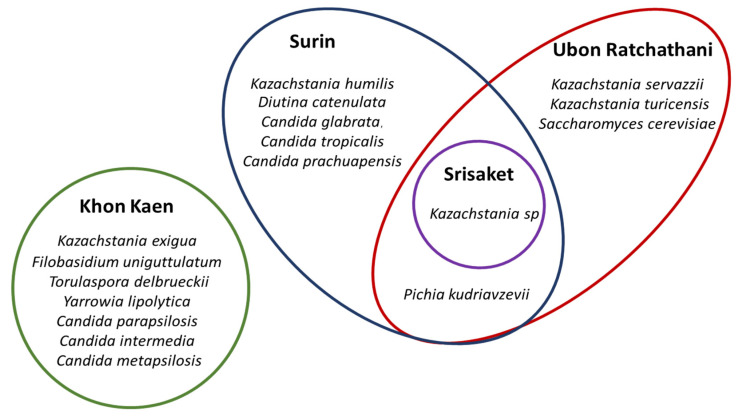
Distribution of the yeast genera in overall Plaa-som samples collected from 4 different fresh markets in Thailand (Venn diagram).

The distribution of the eight yeast genera in all of the Plaa-som samples (*n* = 35) was determined. *Kazachstania* was revealed to be the highest yeast genus distribution yeast in Plaa-som samples. This yeast was detected in samples of Plaa-som collected from all four provinces in this study, shown as a Venn diagram in Figure 6. Furthermore, the frequency of occurrence (%) showed that *Kazachstania* was 40.0% (found in 14 samples) while *Candida* was 20% (found in seven samples). *Yarrowia* was 11.43% (found in four samples), *Pichia* and *Diutina* were 5.71% (found in two samples), while *Filobasidium*, *Torulaspora* and *Saccharomyces* were 2.86% (found in just one sample). This is different from previous research that indicated the most common yeasts in Plaa-som are those of the genera *Candida*, *Saccharomyces*, *Pichia*, and some species of *Zygosaccharomyces*, *Debaryomyces*, and *Hanseniaspora* [6,21,22,33,43].

In Figure 6, the diversity of yeasts found in Plaa-som prepared in Surin, Sisaket, and Ubon Ratchathani were more closely related than that found in Plaa-som prepared in Khon Kaen. The geographical distribution of yeasts may play a role in this finding. Surin, Sisaket, and Ubon Ratchathani are neighboring provinces located at the area called South ISAN (Lower Northeatern Thailand), whereas Khon Kaen is several hundred kilometers north of those provinces. More evidence is required to draw a strong conclusion on this issue. At this point, it is premature to conclude that different communities affect properties, taste, and aroma of Plaa-som because many factors attribute to these characteristics of Plaa-som, especially their unique way of being prepared.

The genus *Kazachstania* is an osmophilic yeast that is normally found in foods high in salt or sugar, such as kimchi, miso, Chinese bean peppers (fermented chili paste), jam, and honey [44,45,46,47]. Furthermore, many yeasts in this genus have been found in fermented foods and beverage, e.g., *Kazachstania hellenica* in fermented grapes [48]; *K. exigua* in fermented onions [49]; *K. exigua*, *K. saulgeensis*, and *K. humilis* in sourdough [50,51,52,53]; and *K. barnettii* in kimchi [53]. However, none of these yeasts were reported in fermented fish. Therefore, our finding is the first to reveal *Kazachstania* as a dominant yeast that is widely distributed in Plaa-som (Thai) fermented fish. Furthermore, it was surprising to discover that all yeast *Kazachstania* sp. isolated from Plaa-som samples were identified as a new species, *Kazachstania surinensis* [54].

Yeasts are responsible for the aroma and flavor of many fermented foods and beverages such as bread, soy sauce, cheeses, beer, wine, and sake. Moreover, *S. cerevisiae* is the most dominant species of yeast used in food fermentation. However, some non-*Saccharomyces* yeasts also play a role in fermented foods such as cheeses, koumiss, soy sauce, fermented sausages, and sourdough bread. These non-*Saccharomyces* yeasts include the so-called dairy yeasts *(Debaryomyces hansenii, Geotrichum candidum, Kluyveromyces lactis*, *K. marxianus*, and *Yarrowia lipolytica*), sourdough yeast (*Kazachstania humilis*), soy sauce yeast (*Zygosaccharomyces rouxii*), and fermented fish (*Z. rouxii* and *Z. baili*), among others. Such yeasts contribute to flavor by converting sugars, amino acids, glycosides, and phenolic acids into alcohols, esters, carboxylic acids, lactones, aldehydes, and other volatile compounds via their fermentation processes [6,50,53,55]. Several researchers had reported beneficial effects on the development of desirable aromas in a plant-based lactic acid fermentation microenvironment by the coexistence of *Kazachstania* yeast and lactic acid bacteria in many fermented foods and beverages [44,45,49,56,57]. However, the adaptive mechanism and metabolic characteristics of these *Kazachstania* yeasts in a lactic acid environment is still unclear. Therefore, the *Kazachstania* yeasts in our study possibly demonstrated significant yeasts that play a role in the development of desirable colors and aromas of fermented fish. However, the precise role of *Kazachstania* in aroma and flavor formation in fermented fish remains to be investigated.

The second most dominant and distributed genus was *Candida*, consisting of three clades: *Candida/Lodderomyces clade* (*C. tropicalis*, *C. rachuapensis*, *C. metapsilosis*, and *C. parapsilosis*), *Candida/Nakaseomyces clade* (*C. glabrata*), and *Candida/Metschnikowia clade* (*C. intermedia*). Our analysis revealed that these *Candida* yeasts were found in fermented fish samples from two provinces (20%). However, some of yeast strains, such as *C. tropicalis*, *C. glabrata*, *C. intermedia*, and *C. parapsilosis*, have been reported as spoilage yeasts and opportunistic human pathogens [58,59,60,61,62]. They may present as a result of unsanitary manufacturing processes. Moreover, the use of raw fish without thermal treatment contributes to the presence of pathogens in the final product [58,59,63,64,65].

For the case of *Diutina catenulata* and *Pichia kudriavzevii,* there are few reports of human infection with *D. catenulata*, and only a small number of studies are available to guide clinical treatment [66]. In addition, *P. kudriavzevii* has the potential of producing a toxin that is able to kill several pathogenic microorganisms, thereby contributing to food preservation [67]. This organism has also been considered a potential probiotic for its ability to assimilate cholesterol [68]. In conclusion, not all strains of *Diutina catenulata* and *Pichia kudriavzevii* appear to be opportunistic pathogens.

In addition to the two reasons mentioned above, yeast contamination in Plaa-som can occur during product transportation, during market sales, or cross-contamination from other fermented food that is sold in the same area. Unfortunately, we cannot be certain as to “why and how” these yeasts occur in Plaa-som due to our research not being related to this topic. We think that the pathogenic yeasts found in Plaa-som cannot harm consumers because this food is not commonly consumed raw but must be cooked by steaming or deep-frying before consumption.

### 3.7. Yeast Community Analysis

From Table 5, the H’ diversity index and Evenness index (J’) for yeast communities in each region were calculated. The H’ values were 1.584, 0.9, 1.237, 0, and 1.438. The high values for H’ represent a more diverse community. However, only the H’ value cannot yet accurately indicate whether each yeast community is more or less diverse. The values of J’ were 0.82, 0.82, 0.89, indeterminate, and 0.89, respectively. The analysis results indicate that each yeast genus was evenly distributed in Plaa-som since the J’ values were close to 1. [69,70,71]. Moreover, the Sorensen’s coefficient similarity index (SCSI) [72] was used for comparative analysis of yeast in each community, the results of which are shown in Table 6.

The SCSI values reported in Table 6 varied from 0.25 to 0.57 or 25 to 57 percent, for each pair of yeast communities from each location. It can be seen that the SCSI values of yeast in Plaa-som in UB-SR and UB-SK were 0.571 and 0.50 (57.1 and 50%), which is higher when compared to the UB-KK (0.25 or 25%). As we mentioned earlier, Ubon Ratchathani, Surin, and Sisaket are neighboring provinces. Therefore, the similarity of yeast in Plaa-som of these three provinces may be due to the use of raw materials (fish, rice, and salt) from sources in the same geographic environment, thus making the microorganisms residing on the raw materials similar. In addition, the traditional fermentation process without sterilization of raw materials combined with the spontaneous fermentation allows indigenous microorganisms from raw materials and environments to be selected and to promote the growth of microorganisms that cause fermentation in foods.

When considering the similarity of yeast in Plaa-som from Khon Kaen Province compared to the three provinces mentioned above, it was found to be between 0.25 to 0.444 (25 to 44%). Khon Kaen is a province located in the north of the Northeastern part of Thailand and is several hundred kilometers from Ubon Ratchathani, Surin, and Sisaket. Therefore, Khon Kaen is geographically different from the three provinces, and there is a possibility that indigenous microorganisms in the raw materials are different. However, more evidence is required to draw a strong conclusion on this issue. At this point, it is too early to conclude that yeast community differences are the result of differences in indigenous microorganisms on raw materials obtained from different geographical sources. Therefore, this point is to be investigated in further research.

**Figure 7:**
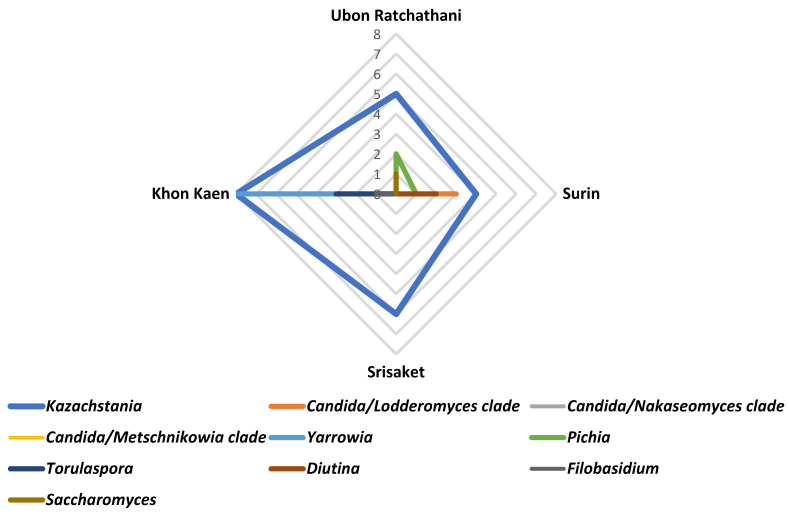
Distribution of yeasts in Plaa-som samples collected from fresh markets located in 4 provinces of Thailand.

The distribution of yeast genera in Plaa-som samples from four provinces was mapped using radar charts (Figure 7). Our findings showed that *Kazachstania* was the most commonly found yeast genus and widely distributed in Plaa-som samples.

## 4. Conclusions

This study demonstrates that Thai fermented fish products (Plaa-som) are primarily produced according to family tradition and local geographic preferences. Therefore, the proportions of raw materials used in the production methods varies from region to region. Differences in these proportions lead to variations in microbial populations, which results in differences in color, aroma, taste, and texture, leading to the uniqueness of Plaa-som products in each area of Thailand. Furthermore, our results also define the distribution and succession of predominant yeast species during Plaa-som fermentation. The predominant yeast species was in the genus *Kazachstania*, consisting of *Kazachstania servazzii*, *K. humilis*, *K. exigual*, *K. turicensis*, and *Kazachstania* sp. Our study is the first report of these yeast species with some differences from previous research papers. This provides useful information for the development of yeast and lactic acid bacteria starter cultures to control the fermentation processes of Plaa-som with the purpose of more consistent quality, texture, and aroma.

## Figures and Tables

**Table 1:** Sources for yeast isolation.

Location	GPS	Collection Date
Warin Municipal fresh market, Prathum Thep Phakdi Road, Warin Chamrap district, Ubon Ratchathani * province	15°11′58.0″ N 104°51′57.1″ E	2 January 2021
Warin Municipal fresh market, Prathum Thep Phakdi Road, Warin Chamrap district, Ubon Ratchathani * province	15°11′58.0″ N 104°51′57.1″ E	3 January 2021
Noi Ruen Rom fresh market, Lak Mueang Road, Mueang Surin district, Surin * province	14°52′50.4″ N 103°29′49.6″ E	14 January 2021
Ton Ma Kluea market, Ratchakan Rodfai 3 Road, Mueang Sisaket, Sisaket * province	15°06′58.0″ N 104°19′56.2″ E	15 January 2021
Municipal fresh market 1, Muang Khon Kaen, Khon Kaen * province	16°25′34.8″ N 102°50′04.3″ E	19 January 2021

* Ubon Ratchathani, Surin, Sisaket, and Khon Kaen provinces are located in the northeast of Thailand.

**Table 2:** The number and percentage of yeast detected and non-yeast detected fresh market samples from different provinces in Thailand.

Provinces	Samples with Detectable Yeasts	Samples without Detectable Yeasts	Total	
			Number of Samples	Percentage of Samples with Detectable Yeasts
Ubon Ratchathani (UB)	4	5	9 (*n*)	44.44
Sisaket (SK)	5	1	6 (*n*)	83.33
Surine (SR)	6	7	13 (*n*)	46.15
Khon Kaen (KK)	5	2	7 (*n*)	71.43
Total	20	15	35 (*n*)	57.14

**Table 3:** Yest species found in Plaa-som samples collected from fresh markets in Thailand.

Species	Number of Isolates	Relative Frequency (%)
*Candida tropicalis* (*Candida*/*Lodderomyces* clade)	2	3.85
*C. metapsilosis* (*Candida*/*Lodderomyces* clade)	1	1.92
*C. parapsilosis* (*Candida*/*Lodderomyces* clade)	3	5.77
*C. prachuapensis* (*Candida*/*Lodderomyces* clade)	1	1.92
*C. intermedia* (*Clavispora* clade)	2	3.85
*C. glabrata* (*Nakaseomyces* clade)	2	3.85
*Diutina catenulata (2)*	2	3.85
*Filobasidium uniguttulatum (1)*	1	1.92
*Kazachstania servazzii*	2	3.85
*K. humilis*	2	3.85
*K. exigual*	8	15.38
*K. turicensis*	2	3.85
*Kazachstania* sp.	9	17.31
*Pichia kudriavzevii (2)*	3	5.77
*Saccharomyces cerevisiae (1)*	1	1.92
*Torulaspora delbrueckii (1)*	3	5.77
*Yarrowia lipolytica*	8	15.38
Total	52	100.00

**Table 4:** Molecular identification of yeasts isolated from Plaa-som samples collected from fresh markets in Thailand.

Isolation Code	Closest Species (GenBank Accession Number)	Nucleotide in D1/D2 Region					Result of Identification
		Identity/ Total	Similarity (%)	No. of Gap	Substitution		
					No	%	
UBFS 11-1	*Kazachstania turicensis* CBS 8665^T^ (NG_058312)	577/578	99.82	0	1	0.17	*Kazachstania turicensis*
UBFS 16-1	*Kazachstania servazzii* NRRL Y-12661^T^ (NG_055029)	591/592	99.83	0	1	0.17	*Kazachstania servazzii*
UBFS 16-2	*Kazachstania turicensis* CBS 8665^T^ (NG_058312)	577/578	99.82	0	1	0.17	*Kazachstania turicensis*
UBFS 16-3	*Kazachstania servazzii* NRRL Y-12661^T^ (NG_055029)	591/592	99.83	0	1	0.17	*Kazachstania servazzii*
UBFS 17-1	*Saccharomyces cerevisiae * NRRL Y-12632^T^ (NG_042623)	592/592	100	0	0	0	*Saccharomyces cerevisiae*
UBPS-1	*Pichia kudriavzevii* CBS 5147^T^ (MH545928)	578/584	100	6	0	0	*Pichia kudriavzevii*
UBPS-5	*Pichia kudriavzevii* CBS 5147^T^ (MH545928)	576/584	99.65	6	2	0.34	*Pichia kudriavzevii*
UBPS-6	*Kazachstania humilis * CBS 5658^T^ (KY106507)	586/590	99.32	0	4	0.68	*Kazachstania* sp. **
SRFS 42-1	*Diutinaatenulate* CBS 565^T^ (MK394156)	485/485	100	0	0	0	*Diutina catenulate*
SRFS 42-2	*Kazachstania humilis* CBS 5658T (KY106507)	589/590	99.83	0	1	0.17	*Kazachstania humilis*
SRFS 43-1	*Kazachstania humilis* CBS 5658^T^ (KY106507)	588/590	99.66	0	2	0.33	*Kazachstania humilis*
SRFS 45-1	*Candida prachuapensis * CBS 11024^T^ (NG_054767)	570/570	100	0	0	0	*Candida prachuapensis*
SRFS 47-1	*Diutinaatenulate* CBS 565^T^ (MK394156)	484/485	99.79	0	1	0.21	*Diutina catenulata*
SRFS 56-1	*Pichia kudriavzevii * CBS 5147^T^ (MH545928)	583/584	99.82	0	1	0.18	*Pichia kudriavzevii*
SRFS 56-2	*Candida glabrata* CBS 138^T^ (MH545922)	599/601	99.66	0	2	0.33	*Candida glabrata*
SRFS 56-3	*Kazachstania humilis* CBS 5658^T^ (KY106507)	585/591	99.15	1	5	0.85	*Kazachstania* sp. *
SRFS 56-7	*Candida tropicalis * CBS 94^T^ (KY106838)	551/552	100	1	0	0	*Candida tropicalis*
SRFS 57-1	*Candida tropicalis * CBS 94^T^ (KY106838)	551/552	100	1	0	0	*Candida tropicalis*
SRFS 57-2	*Kazachstania humilis* CBS 5658^T^ (KY106507)	585/591	99.15	1	5	0.85	*Kazachstania* sp. *
SRFS 57-3	*Candida glabrata* CBS 138^T^ (MH545922)	599/601	99.66	0	2	0.33	*Candida glabrata*
SKFS 62-1	*Kazachstania humilis* CBS 5658^T^ (KY106507)	585/590	99.15	0	5	0.85	*Kazachstania* sp. *
SKFS 63-1	*Kazachstania humilis* CBS 5658^T^ (KY106507)	585/590	99.15	0	5	0.85	*Kazachstania* sp. **
SKFS 63-2	*Kazachstania humilis* CBS 5658^T^ (KY106507)	585/590	99.15	0	5	0.85	*Kazachstania* sp. **
SKFS 65-1	*Kazachstania humilis* CBS 5658^T^ (KY106507)	586/590	99.32	0	4	0.67	*Kazachstania* sp. **
SKFS 66-1	*Kazachstania humilis* CBS 5658^T^ (KY106507)	585/590	99.15	0	5	0.85	*Kazachstania* sp. *
SKFS 67-1	*Kazachstania humilis* CBS 5658^T^ (KY106507)	585/590	99.15	0	5	0.85	*Kazachstania* sp. *
KKFSJ 1-1	*Kazachstania exigua* CBS 379^T^ (NG_055049)	588/589	99.83	0	1	0.17	*Kazachstania exigua*
KKFSJ 1-2	*Kazachstania exigua* CBS 379^T^ (NG_055049)	587/589	99.66	0	2	0.33	*Kazachstania exigua*
KKFSJ 1-3	*Yarrowia lipolytica* CBS 6124^T^ (MH545931)	524/525	100	1	0	0	*Yarrowia lipolytica*
KKFSP 1-1	*Kazachstania exigua* CBS 379^T^ (NG_055049)	588/589	99.83	0	1	0.17	*Kazachstania exigua*
KKFSP 1-2	*Kazachstania exigua* CBS 379^T^ (NG_055049)	588/589	99.83	0	1	0.16	*Kazachstania exigua*
KKFSP 1-3	*Kazachstania exigua* CBS 379^T^ (NG_055049)	588/589	99.83	0	1	0.16	*Kazachstania exigua*
KKFSP 1-4	*Kazachstania exigua* CBS 379^T^ (NG_055049)	588/589	99.83	0	1	0.16	*Kazachstania exigua*
KKFSP 1-5	*Kazachstania exigua* CBS 379^T^ (NG_055049)	588/589	99.83	0	1	0.17	*Kazachstania exigua*
KKFSP 1-6	*Kazachstania exigua* CBS 379^T^ (NG_055049)	589/591	100	2	0	0	*Kazachstania exigua*
KKFSP 1-7	*Candida parapsilosis * CBS 604^T^ (MH545914)	587/590	100	3	0	0	*Candida parapsilosis*
KKFSP 1-8	*Yarrowia lipolytica* CBS 6124^T^ (MH545931)	523/525	99.8	1	1	0.2	*Yarrowia lipolytica*
KKFSP 2-1	*Filobasidium uniguttulatum * CBS 1730^T^ (KY107727)	532/534	100	2	0	0	*Filobasidium uniguttulatum*
KKFSP 2-2	*Candida parapsilosis* CBS 604^T^ (MH545914)	589/590	99.83	0	1	0.17	*Candida parapsilosis*
KKFSP 2-3	*Torulaspora delbrueckii * CBS 1146^T^ (NG_058413)	593/593	100	0	0	0	*Torulaspora delbrueckii*
KKFSP 2-4	*Torulaspora delbrueckii * CBS 1146^T^ (NG_058413)	592/593	99.83	0	1	0.17	*Torulaspora delbrueckii*
KKFSP 2-5	*Torulaspora delbrueckii * CBS 1146^T^ (NG_058413)	592/593	99.83	0	1	0.17	*Torulaspora delbrueckii*
KKFSC 1-1	*Yarrowia lipolytica* CBS 6124^T^ (MH545931)	521/526	100	5	0	0	*Yarrowia lipolytica*
KKFSC 1-2	*Candida intermedia * CBS 572^T^ (AY497683)	350/353	99.71	2	1	0.19	*Candida intermedia*
KKFSC 1-3	*Yarrowia lipolytica* CBS 6124^T^ (MH545931)	523/525	99.8	1	1	0.19	*Yarrowia lipolytica*
KKFSC 1-4	*Yarrowia lipolytica* CBS 6124^T^ (MH545931)	521/525	100	4	0	0	*Yarrowia lipolytica*
KKFSC 1-5	*Candida intermedia * CBS 572^T^ (AY497683)	348/354	99.14	3	3	0.85	*Candida intermedia*
KKFSC 1-6	*Candida metapsilosis * CBS 10907^T^ (MK394127)	589/590	99.83	0	1	0.16	*Candida metapsilosis*
KKFSG 1-1	*Yarrowia lipolytica* CBS 6124^T^ (MH545931)	523/524	100	1	0	0	*Yarrowia lipolytica*
KKFSG 1-2	*Yarrowia lipolytica* CBS 6124^T^ (MH545931)	523/524	99.8	0	1	0	*Yarrowia lipolytica*
KKFSG 1-3	*Candida parapsilosis * CBS 604^T^ (MH545914)	589/590	99.83	0	1	0	*Candida parapsilosis*
KKFSG 1-4	*Yarrowia lipolytica* CBS 6124^T^ (MH545931)	524/525	100	1	0	0	*Yarrowia lipolytica*

* The strains were proposed as a new species, namely *Kazachtania surinensis* [42]; ** The strains were identified as *K. surinensis* that was discovered after the new species description.

**Table 5:** Yeast community analysis.

Diversity Indices	Overall	UB	SR	SK	KK
Shannon–Wiener index (*H’*)	1.584	0.9	1.237	0	1.438
Evenness index (J’)	0.82	0.82	0.89	indet	0.89

UB, Ubon Ratchathani; SR, Surin; SK, Sisaket; KK, Khon Kaen; indeterminate; indeterminate.

**Table 6:** The Sorensen’s coefficient similarity index (SCSI) of the yeast community.

Yeast Community	Sorensen’s Coefficient Similarity Index (SCSI)
UB-SR	0.571
UB-SK	0.50
UB-KK	0.25
SR-SK	0.40
SR-KK	0.444
SK-KK	0.333

UB, Ubon Ratchathani; SR, Surin; SK, Sisaket; KK, Khon Kaen.

## Data Availability

All sequence data are available in NCBI GenBank following the accession numbers in the manuscript.
